# Randomized, Prospective Double-Blinded Study Comparing 3 Different Doses of 5-Aminolevulinic Acid for Fluorescence-Guided Resections of Malignant Gliomas

**DOI:** 10.1093/neuros/nyx074

**Published:** 2017-04-01

**Authors:** Walter Stummer, Herbert Stepp, Otmar D. Wiestler, Uwe Pichlmeier

**Affiliations:** * Department of Neurosurgery, University of Münster, Münster, Germany; ‡ Laser-Research Laboratory, LIFE-Center at University Hospital of Munich, Munich, Germany; § German Cancer Research Center (DKFZ), Heidelberg, Germany; ¶ Medac GmbH, Gesellschaft für klinische Spezialpräparate mbH, Wedel, Germany

**Keywords:** Fluorescence-guided resections, Malignant glioma, 5-ALA, Aminolevulinic acid, Randomized study, Spectrography, Histology

## Abstract

**BACKGROUND:** Five-aminolevulinic acid (5-ALA) is used for fluorescence-guided resections of malignant glioma at a dose of 20 mg/kg; yet, it is unknown whether lower doses may also provide efficacy.

**OBJECTIVE:** To perform a double-blinded randomized study comparing 3 different doses of 5-ALA.

**METHODS:** Twenty-one patients with suspected malignant glioma were randomly assigned to 0.2, 2, or 20 mg/kg 5-ALA. Investigators were unaware of dose. Intraoperatively, regions of interest were first defined in tumor core, margin, and adjacent white matter under white light. Under violet–blue illumination, the surgeon's impression of fluorescence was recorded per region, followed by spectrometry and biopsy. Plasma was collected after administration and analyzed for 5-ALA and protoporphyrin IX (PPIX) content.

**RESULTS:** The positive predictive value of fluorescence was 100%. Visual and spectrometric fluorescence assessment showed 20 mg/kg to elicit the strongest fluorescence in tumor core and margins, which correlated with cell density. Spectrometric and visual fluorescence correlated significantly. A 10-fold increase in 5-ALA dose (2-20 mg/kg) resulted in a 4-fold increase of fluorescence contrast between marginal tumor and adjacent brain. t_max_ for 5-ALA was 0.94 h for 20 mg/kg (0.2 kg: 0.50 h, 2 mg/kg: 0.61 h). Integrated PPIX plasma levels were 255.8 and 779.9 mcg*h/l (2 vs 20 mg/kg). Peak plasma concentrations were observed at 1.89 ± 0.71 and 7.83 ± 0.68 h (2 vs 20 mg/kg; average ± Standard Error of Mean [SEM]).

**CONCLUSION:** The highest visible and measurable fluorescence was yielded by 20 mg/kg. No fluorescence was elicited at 0.2 mg/kg. Increasing 5-ALA doses did not result in proportional increases in tissue fluorescence or PPIX accumulation in plasma, indicating that doses higher than 20 mg/kg will not elicit useful increases in fluorescence.

**ABBREVIATIONS**
5-ALA: five-aminolevulinic acid
AUC: area under curve
b.w.: body weight
CNS: central nervous system
GBM: glioblastoma multiforme
KPS: Karnofsky Performance Scale
NIH: National Institute of Health
PPIX: protoporphyrin IX
SD: standard deviation
SEM: Standard Error of Mean

**F**ive-aminolevulinic acid (5-ALA) elicits the accumulation of fluorescent protoporphyrin IX (PPIX) in malignant glioma tissue and is used for fluorescence-guided resections.^1-18^

However, little is known regarding PPIX pharmacokinetics after administration of 5-ALA, which is employed at a dose of 20 mg/kg body weight (b.w.) for brain tumor surgery. The use of lower doses of 5-ALA for brain tumor surgery has been reported. For example, Haj-Hosseini et al^19,20^ administered only 5 mg/kg b.w. 5-ALA for detecting tumor fluorescence using a touch pointer with a diode laser light source and 405 nm light for fluorescence excitation. We have used 10 mg/kg in our initial experience but felt 20 mg/kg to be better without providing firm evidence.^4,8^ Thus, it is unknown whether lower doses of 5-ALA might be equally efficacious for eliciting visually perceivable fluorescence and where the lower threshold for a useful dosage is.

5-ALA is not without systemic side effects, especially at high doses, at which nausea, vomiting, and cardiovascular reactions have been noted.^21-23^ Even at 20 mg/kg, patients may develop skin phototoxicity and a significant but temporary increase in liver enzymes.^5^ Therefore, lower doses of 5-ALA might be preferable. Here we address the question of whether lower doses of 5-ALA elicit enough fluorescence for macroscopic detection of residual tumor, ie, through the operating microscope. Due to the many confounders such a study might have, we have designed a randomized, double-blind study investigating 0.2, 2, and 20 mg/kg b.w. of 5-ALA, determining fluorescence accumulation both macroscopically and spectrometrically, correlating these findings with tumor cell density, and determining the usefulness of the observed fluorescence for supporting fluorescence-guided resections. A secondary focus was on toxicological side effects.

## METHODS

This prospective, single-center, double-blind, randomized, 3-dose level phase I/II trial was performed in accordance with good clinical practice and sponsored by Medac, Hamburg, Germany. The study was approved by the Independent Ethics Committee of the Ludwig-Maximilian University Munich, Germany (Project-No. 029/00) and is registered at clinicaltrials.gov (ClinicalTrials.gov Identifier: NCT02755142). The dose levels 0.2 and 2 mg/kg of 5-ALA are below the approved dose in Europe and thus off label. No approval for 5-ALA is available in the United States as yet.

The study included males and females aged 18 to 75 years, a Karnofsky Performance Scale (KPS) score of ≥70%, with magnetic resonance imaging suggestive for malignant gliomas WHO grade III or IV, for whom surgical treatment was indicated and who had had no other operation or tumor-specific pretreatments. Patients with porphyria or hypersensitivity to porphyrins, renal or hepatic insufficiency, or malignomas (except for basaliomas) were excluded. Each patient's written informed consent was required.

All patients received 3 × 4 mg of dexamethasone at least 2 days prior to surgery.

### Study Objectives

The primary study objective was to detect a dose–efficacy relationship between drug dose and overall tumor fluorescence as perceived by the surgeon under the operating microscope (NC4 OPMI Fluoro, Carl Zeiss, Oberkochen, Germany). For tumor resection, the surgeon could alternate freely between white and blue illumination. After resection of necrotic tumor regions, the global fluorescence impression of residual solid core tumor (as identified under white light illumination) was determined. It was estimated whether approximately 0/3, 1/3, 2/3, or 3/3 of the tumor core as identified under white light revealed fluorescence (irrespective of fluorescence quality). In addition, the surgeon assessed the general fluorescence quality in the tumor core on a 3-tiered scale (“strong,” “weak,” or “no” fluorescence).

**FIGURE 1.**
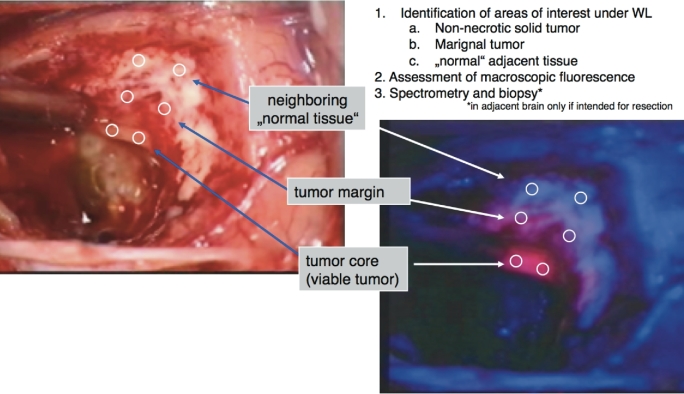
Procedure for sampling and spectrometry. In a first step, after removal of necrotic tumor, regions of interest were identified under white light consisting of non-necrotic solid tumor (core), marginal tumor, and normal adjacent tissue under normal white light illumination. Second, the macroscopic fluorescence quality was assessed by the surgeon in each region (“strong,” “weak,” “none”), immediately followed by spectrometry in the respective region and biopsies. In “normal” adjacent tissue, biopsies were only collected if this tissue was intended for resection.

Secondary objectives encompassed spectrographic measurements of fluorescence in 3 predefined tissue regions: viable (non-necrotic) tumor core, marginal tumor, and adjacent, normally appearing tissue. These measurements were correlated with the macroscopic fluorescence impression and histology (Figure 1; Table 1). In detail, 3 different tissue regions were identified under white light and the microscope subsequently switched to blue light. Macroscopic fluorescence impressions were recorded in the predefined regions, followed by spectrographic measurements at 2 sites, which were averaged, followed by 1 biopsy in each region. If different fluorescence qualities (weak, strong, or no fluorescence) were perceived in a single-tissue region, these qualities were assessed individually, ie, with 2 spectrographic measurements and 1 biopsy each.

**TABLE 1.** Overview of Strategy for Intraoperative Tissue Sampling and Spectrometry

Location	Fluorescence quality	Selection of areas	Spectrometry	Biopsy
Tumor core	Strong	2	1× per area	1
	Weak	2	1× per area	1
	None	2	1× per area	1
Tumor margin	Strong	2	1× per area	1
	Weak	2	1× per area	1
	None	2	1× per area	1
Normal tissue	None	3	1× per area	1 (optional)

Theoretical maximum number of measurements. Two spectra were obtained from each selected area and averaged. Biopsies in “normal” tissue were only collected if this tissue was intended for resection.

Three measurements were obtained from adjacent brain (which were averaged) and biopsied only if that part of the brain was intended for resection. Because this was a double-blinded, randomized study and it was unknown to what extent fluorescence would be visible in a given patient, it was not permissible to define the areas for spectrographic measurement by the fluorescence impression.

Spectrography was performed in Situ using the D-Light System (Karl Storz, Tuttlingen, Germany) coupled to a fiber probe array consisting of a centrally arranged 400-μm detection fiber surrounded by 6400-μm excitation fibers (TuiLaser AG, Munich, D), as previously described.^9^ The detection fiber was coupled to an S2000 spectrometer (Ocean Optics, Eerbeek, the Netherlands). Excitation light was filtered by a longpass filter (435 nm) to block reflected excitation light. Measurements were performed in freshly exposed tissue to minimize fluorophore degeneration by photobleaching. Three spectra were obtained at each individual site and averaged. All spectra were normalized relative to a fluorescent, nonbleaching reference object that fluoresced in the range of the porphyrin spectrum. Reference measurements were obtained prior to each tissue measurement to account for short-term fluctuations in excitation light.^9^

PPIX exhibits characteristic peaks at 635 nm, which is in the visible red range, and a smaller peak at 704 nm. The latter peak is in the near infrared range and not clearly visible to the human eye. For this reason, the 635-nm peak was used for quantifying fluorescence. To closely approximate visually perceived fluorescence, no corrections were performed for tissue autofluorescence background, absorption, or scattering.

### Histology

Biopsies were analyzed for cell density by estimating the range (min/max) of densities on each histological section. For statistical evaluations, the arithmetic mean of the biopsy-specific minima and maxima was calculated. These means were used to calculate the arithmetic mean of the biopsy-specific means within each fluorescence quality, and within each location (tumor core and tumor margin) for each patient. Histological assessments were performed centrally by one of the authors who was blinded to study group allocation (OW).

### Pharmacokinetic Investigations

Concentrations of 5-ALA and PPIX were determined in plasma at 0 (predose), 15, 30, 45, 60, and 90 min and 2, 3, 4, 5, 6, 7, 8, 10, 12, 18, 24, and 48 h after administration by high-performance liquid chromatography (IKP GmbH, Grünstadt, Germany), and model-independent pharmacokinetic parameters were calculated using noncompartmental methods as follows:

- area under data from time zero to last quantifiable concentrations (AUC_0-t_),
- apparent terminal elimination half-life (t_1/2λz_),
- area under data from time zero to infinity (AUC_0-inf_) = AUC_0-t_ + C_t_/λ_z_,
- concentration maximum (C_max_),
- time of concentration maximum (t_max_).

### Safety Assessments

Adverse events were evaluated using the National Cancer Institute Common Toxicity Criteria. Safety monitoring included laboratory assessments (hematology, liver and renal function, and biochemistry), cardiological parameters (blood pressure, pulse, electrocardiogram), and functional measures (KPS, National Institute of Health [NIH] stroke score).

### Randomization

Block randomization was performed by opening a preprepared sealed randomization envelope after a patient's registration.

Supplies of 5-ALA (Medac GmbH, Wedel, Germany) were dispensed by the hospital pharmacy according to patient weight and placed in closed, nontransparent containers to be indiscernible to the study staff. The ready-for-use solution was prepared by dissolving the test drug with 50 mL of water injected into the light-sealed drinking container without opening the container. Patients received 5-ALA at 0.2, 2, or 20 mg/kg b.w. 3 h (range 2.5-3.5 h) prior to anesthesia.

For statistical methods, please refer to **Supplemental Digital Content 1**.

## RESULTS

### Patient Demographics

All 21 randomized patients underwent tumor resection and completed 28 days of follow-up. Patients in the different dose groups were comparable for age, sex, KPS, NIH stroke score, and weight. Eighteen patients had glioblastomas, 2 patients had gliosarcomas, and 1 patient an anaplastic astrocytoma (Table 2).

**TABLE 2.** Patient Characteristics; No Significant Differences were Noted

Dose (mg/kg b.w.)	0.2	2	20	All
n	7	7	7	21
Age				
Median	59	63	62	59
Min	57	51	37	37
Max	65	70	69	70
Mean	59.7	61.3	56.0	59.0
SD	2.5	6.9	13.5	8.7
Sex				
Male	4 (57%)	4 (57%)	6 (86%)	14(67%)
Female	3 (43%)	3 (43%)	1 (14%)	7 (33%)
Weight (kg)				
Median	70	73	74	72.5
Min	66	52	62	52
Max	102	101.5	82	102
Mean	75.81	78.79	72.86	75.82
SD	12.8	19.68	6.54	13.58
KPS				
Min	80	70	70	70
Median	90	90	90	90
Max	90	90	90	90
NIH				
Min	0	0	0	0
Med	1	1	1	1
Max	3	2	1	3
Histology				
AA	0	0	1	1
Glioblastoma	7	5	6	18
Gliosarcoma	0	2	0	2

### Primary Objectives

**FIGURE 2.**
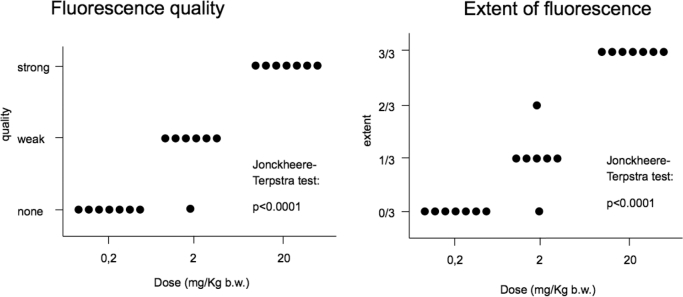
Surgeons’ subjective perception of global fluorescence quality and extent in identifiable tumor (under white light), stratified by dose. “Extent” is defined as the area of fluorescing tissue relative to the area of abnormal tissue as identified under white light illumination.

Overall, macroscopic fluorescence in the tumor core was highest in the 20 mg/kg group, and in all cases the surgeon estimated the entire viable tumor core to fluoresce. With 2 mg/kg, fluorescence was generally perceived as weak, and not the entire tumor core was found to fluoresce. In 1 case, no fluorescence was noted. No fluorescence was perceived in patients in the 0.2 mg/kg group (Figure 2), indicating a monotone, nonfalling dose–efficacy relationship (Jonckheere–Terpstra test: *P* < .0001). Furthermore, all 3 dose groups differed significantly with respect to fluorescence quality and extent of fluorescence (Wilcoxon–Mann–Whitney test), with 20 mg/kg b.w. being the most effective within the tumor core.

**FIGURE 3.**
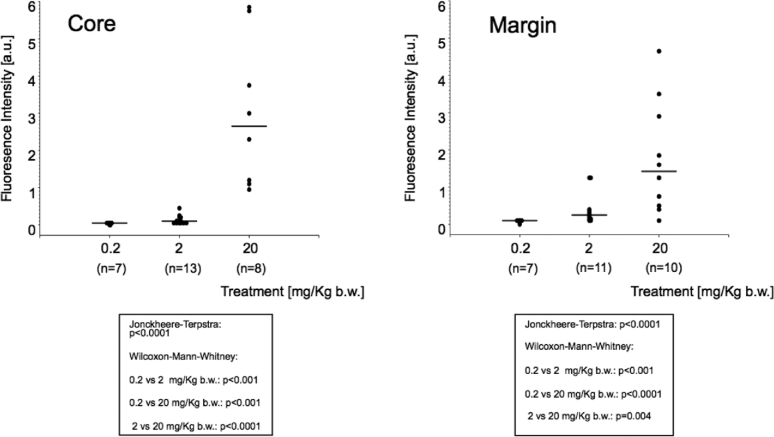
Spectrometrical assessment of fluorescence as measured in the region identified as tumor “core” and “margin” under white light, stratified by 5-ALA dose. Values for each fluorescence quality in single patient averaged.

Similar results were obtained when analyzing fluorescence spectrographically (Figure 3). Twenty milligram 5-ALA per kg b.w. induced the strongest fluorescence in the tumor core as well as in the tumor margins again with a monotone, nonfalling dose–efficacy relationship (Jonckheere–Terpstra test: *P* < .0001) and with significant differences between groups, as tested with the Wilcoxon–Mann–Whitney test.

### Secondary Objectives

**FIGURE 4.**
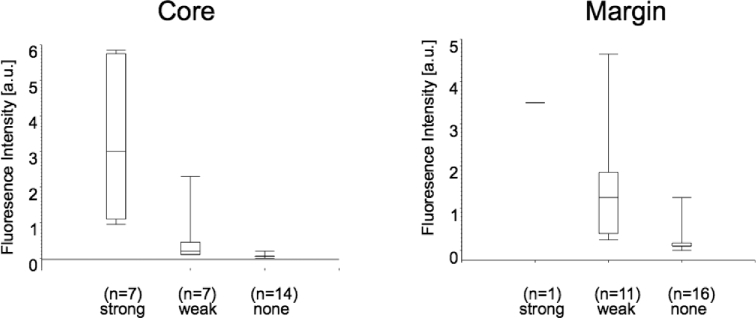
Mean fluorescence intensities as measured spectrometrically vs fluorescence quality (strong, weak, none) in tumor core. Box plots signify minimum, first quartile, median, third quartile, and maximum. Jonckheere–Terpstra test: *P* < .0001; Wilcoxon–Mann–Whitney test: none vs weak fluorescence: *P* = .0001; weak vs strong: *P* = .002; none vs strong: *P* < .0001.

The subjective fluorescence impression (strong, weak, none) correlated significantly with spectrometrically determined fluorescence (Figure 4). However, there was some overlap, indicating macroscopic perception to sometimes differ from spectrographic fluorescence. Strong fluorescence was mostly perceived in the tumor core, as identified under white light, whereas weak fluorescence was mostly found at the tumor margin.

Ratios based on spectrographic fluorescence intensities were determined between tumor core or marginal tumor and normal adjacent brain (Table 3). These ratios were felt to be an indicator of the contrast between tumor and adjacent brain and therefore of importance for discrimination. The ratios between regions with weak and strong fluorescence and normal brain were highest in the group with 20 mg/kg.

**TABLE 3.** Spectrometrically Determined Fluorescence (in arbitrary units, averages ± Standard Error of Mean [SEM]) by Dose and Region (Tumor Core, Tumor Margin, or “Normal” Tissue, as Identified Under White Light Illumination), Normalized to the Fluorescence of a Fluorescence Dummy. R_normal brain_ is the Fluorescence Contrast Ratio Between Tumor Core or Margin Relative to Surrounding Brain. The Ratios are Highest With 20 mg/kg of 5-ALA (SEMs for R Calculated by Error Propagation From SEMs of Fluorescence in the Different Tissue Regions)

Dose (mg/kg)	Core	R_normal brain_	Margin	R_normal brain_	Normal
0.2	0.031 ± 0.005	0.251 ± 0.064	0.077 ± 0.012	0.631 ± 0.164	0.1223 ± 0.026
2	0.129 ± 0.033	0.677 + 0.262	0.417 ± 0.130	2.182 ± 0.933	0.191 ± 0.056
20	2.99 ± 0.708	14.121 ± 5.14	1.75 ± 0.471	8.265 ± 3.19	0.211 ± 0.058

We noted fluorescence to be higher in the single-core samples from the single grade III tumor with a value of 5.86 a.u. than in the samples taken from our glioblastoma multiforme (GBM) patients (mean: 2.38 a.u ± 1.844 standard deviation [SD], 95% CI 1.161 a.u.). Thus, the value for the grade III tumor was outside the 95% SD of the GBMs. However, with this single case it was not possible to draw definite conclusions.

### Histological Examinations

**FIGURE 5.**
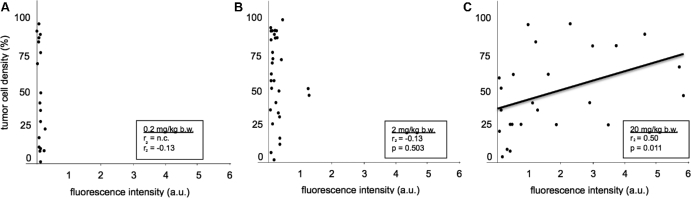
Mean tumor cell density of biopsies vs mean fluorescence intensities as measured spectrometrically, stratified by 5-ALA dose (all localizations including normal brain, all fluorescence qualities, values for single fluorescence qualities in single patient averaged). **A**, 0.2 mg/kg b.w. **B** 2 mg/kg b.w. **C** 20 mg/kg b.w. We only found a relationship between tumor cell density and fluorescence for the 20 mg/kg group. The mean fluorescence intensities per treatment group were 0.767 ± 0.0564 a.u. (0.2 mg/kg); 0.245 ± 0.340 a.u. (2 mg/kg); and 1.71 ± 1.65 a.u. (20 mg/kg; *P* < .0001 by analysis of variance).

A significant positive correlation (Spearman Correlation Coefficient 0.5, *P* = .011) between tumor cellularity and spectrometrically determined fluorescence intensity was observed for the 20 mg/kg b.w. group, but not for the lower dose groups (Figure 5). All tissue samples with macroscopic and/or spectrographic fluorescence showed tumor down to a cellularity of 25% (Figure 5). However, a wide range of fluorescence intensities was detected, even for comparable tumor cell densities. Furthermore, a number of samples with histologically detectable tumor was found, even with high cellularity, with only minimal fluorescence in the 20 mg/kg group, underlining the punctual heterogeneity of fluorescence accumulation.

### Pharmacokinetics

**FIGURE 6.**
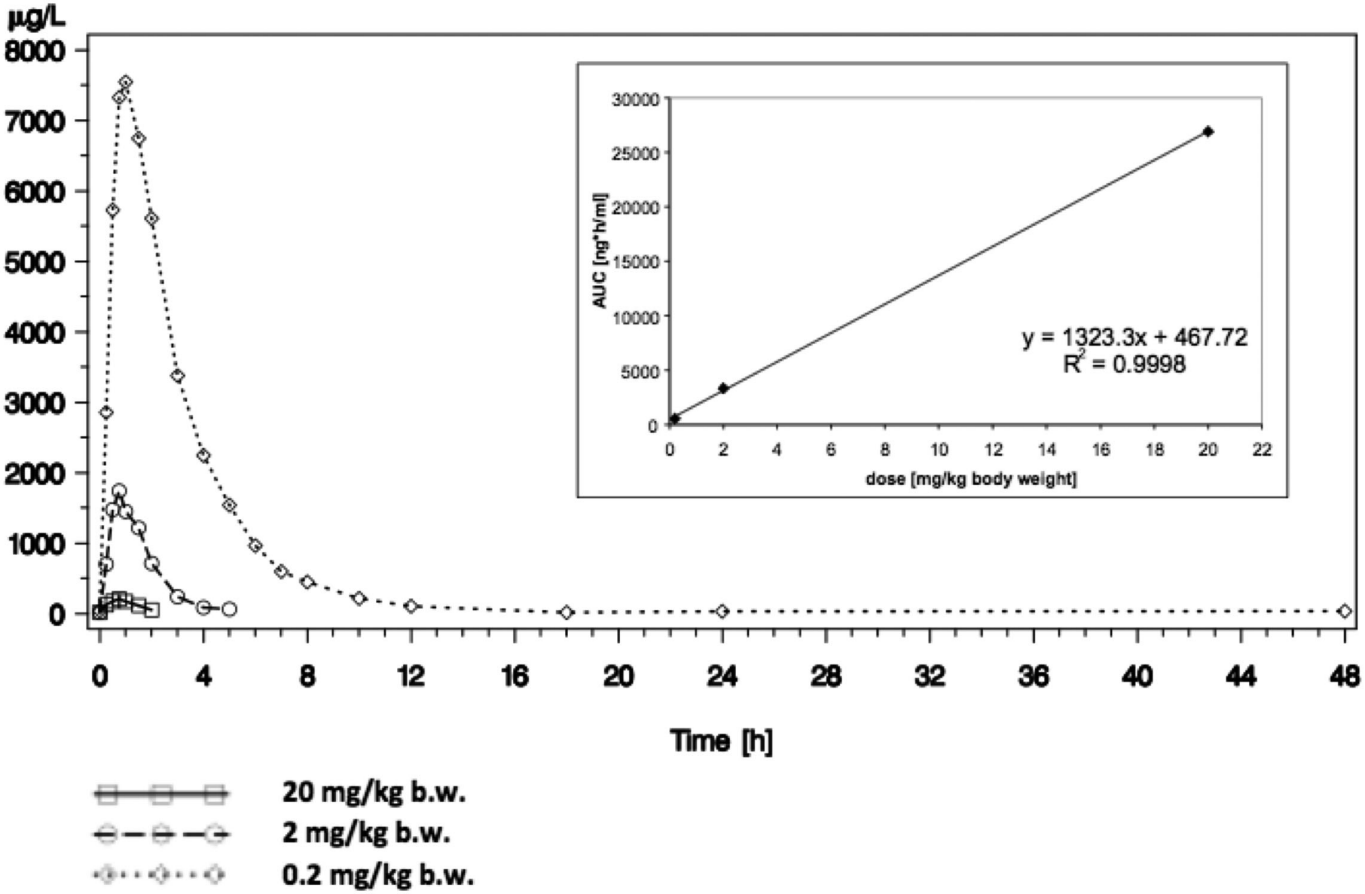
Geometric mean concentrations of 5-ALA [μg/L] vs time [h] for 3 different doses. Inset: dose proportionality of area under curve (AUC) of 5-ALA p.o. in the dose range of 0.2 to 20 mg/kg b.w.

5-ALA was rapidly absorbed after oral administration with t_max_ geometric mean values of 0.50 h for an oral dose of 0.2 mg/kg b.w., 0.61 h for an oral dose of 2 mg/kg b.w., and 0.94 h for an oral dose of 20 mg/kg b.w., respectively. ALA was quickly eliminated with a terminal half-life between 0.85 and 3.05 h. The decrease of the terminal elimination half-life with lower doses was caused by the fact that with lower doses the 5-ALA plasma concentrations became low and the terminal elimination phase could not be captured by sufficient concentrations above the lower limit of quantification of the bioanalytical method, and thus the measured profiles did not represent the terminal elimination rate constants λ_z_ (Figure 6).

**FIGURE 7.**
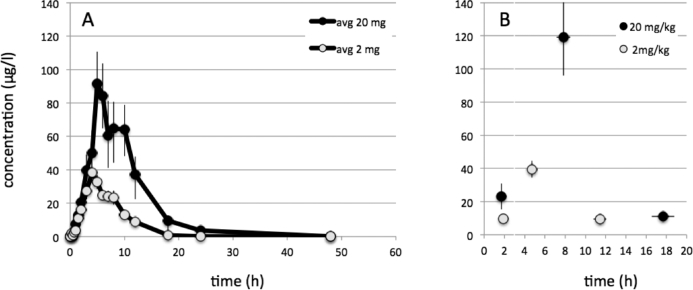
**A**, Geometric mean concentrations of protoporphyrin-IX (PPIX) [μg/L] vs time [h] for 2 and 20 mg/kg averaged at each time point. No PPIX was detectable for the lowest dose of 0.2 mg/kg. **B**, First, peak, and final values for tissue PPIX averaged for each patients with time point of measurement (averages ± SEM) for 2 and 20 mg/kg b.w. On the average, plasma PPIX peaked at 7.8 after 20 mg/kg b.w. and at 4.7 h after 2 mg/kg b.w.

Increased plasma levels of PPIX were observed after 2 and 20 mg/kg b.w., but not with 0.2.mg/kg b.w. Geometric mean area under curve (AUC) values were 255.8 and 779.9 mcg*h/l for 2 and 20 mg/kg b.w., respectively; thus, showing a comparably small 3-fold increase of PPIX after a 10-fold increase 5-ALA. Individual patient peaks were variable. For this reason, individual peaks and the times of their appearance were averaged, showing an average maximum PPIX peak at 7.8 h after 20 mg/kg b.w. and 4.7 h after 2 mg/kg b.w. (Figures 7A and 7B). No PPIX was detected in plasma after 0.2 mg/kg b.w.

For toxicology results, please refer to **Supplemental Digital Content 2**.

## DISCUSSION

### Rationale and Choice of Doses

Fluorescence-guided resections using 5-ALA have expanded the surgical armamentarium for malignant gliomas after drug approval based on the results of a randomized phase III study.^5^ Since then, numerous reports have further explored this technique not only in the context of malignant glioma surgery,^1-18,24^ but also for other tumors, such as meningiomas.^25-27^

The timing of administration of 5-ALA (2.5-3.5 h prior to surgery) has been adapted for ongoing neurosurgical patient studies, as was the dose of 20 mg/kg b.w. The timing of drug administration was initially derived from animal experiments,^7^ in which peak tumor fluorescence was noted at 6 h. The dose of 20 mg/kg, however, was chosen empirically. Higher doses (30-60 mg/kg b.w.) weight have not been used for central nervous system (CNS) tumors and have been reported to have side effects, eg, skin reactions, gastrointestinal symptoms, hemodynamic changes, and increases in liver enzymes.^21-23,28,29^

In the first published experience,^8^ a lower dose of 10 mg/kg b.w. appeared to elicit useful tumor fluorescence. After the first series of patients had been operated on successfully, we increased the dose to 20 mg/kg b.w.^4^ hoping to evoke more fluorescence and increase contrast to normal tissue. The fluorescence yield from 20 mg/kg was felt to be sufficient, and no additional increments were considered. We did not perform a systematic comparison of 10 and 20 mg/kg b.w. at the time, and with the earlier study we did not obtain spectrometrical measurements of fluorescence to compare earlier patients to the present cohort. Furthermore, the microscope used for the early cohort was a prototype modified from an OPMI 2 with different optical characteristics from our present microscope.

On the other hand, others have reported useful, albeit spectrographically detected fluorescence with 5 mg/kg b.w.,^19,20^ and it is unknown whether lower doses may be equally efficacious, in theory avoiding some of the side effects reported for 20 mg/kg (eg, temporary skin phototoxicity or elevated liver enzymes).

The present study provides a more systematic approach for answering the question of whether lower doses of 5-ALA might elicit useful fluorescence. An answer to this question is not easily given, as many confounders exist that render such a study difficult, eg, inherent heterogeneity in the fluorescence.^15,30,31^ Second, it is unclear how the surgically relevant levels of fluorescence should be determined for such a study. There are objective strategies for quantifying porphyrins and the ensuing fluorescence, such as extraction of porphyrins or by spectrometry with corrections for absorption or scattering.^25,30,32^ However, these assessments may give different results compared to the subjective, visual impression of the neurosurgeon using the microscope due to factors such as illumination geometry and tissue characteristics.^25^

Also, on a microscopic level, there is marked heterogeneity of fluorescence levels in tumor tissue, as again demonstrated in this study (Figure 5). The influence of fluorescence heterogeneity, illumination geometry, and tissue characteristics lead us to (a) include objective measures, such as spectrometry and semiquantitative blinded histology; (b) perform a randomized, double-blinded study; and (c) to choose exponential rather than linear dosage increments to avoid underpowering the study, reasoning that smaller increments would not allow the detection of significant differences due the many variables influencing fluorescence.

Higher doses than 20 mg/kg b.w. were not considered due to the previously described side effects. In retrospect, this decision was well justified, because we found a 10-fold increase in ALA dose (2-20 mg/kg) to result in only a 4-fold increase in the fluorescence ratio or contrast between marginal tumor and adjacent brain (Table 3). Discrimination of marginal tumor from normal brain is the most decisive advantage of fluorescence-guided resections.

Correspondingly, our pharmacokinetic analyses demonstrated that the AUC of PPIX in plasma also only increased by a factor of 3 with the 10-fold 5-ALA dose, and the maximal concentration of PPIX increased by a factor of 3.7. The comparably weak increase in PPIX yields was not the consequence of less bioavailability of 5-ALA at higher doses. The AUC for 5-ALA was almost proportional to the applied dose. Thus, it is unlikely that exposure to more 5-ALA simply translates into more PPIX. Rather, the capacity of PPIX accumulation as a response to excess exogenous 5-ALA appears restricted. This was our past experience^7,33^ and the experience of others^34^ with cell lines incubated continuously in 5-ALA. In these experiments, fluorescence began to plateau after 120 min. Heme biosynthesis under physiological circumstances is governed by specific feedback inhibition of ALA synthetase. As this step is overcome by simply administrating excess 5-ALA, physiological inhibition does not explain the restrictions to this accumulation. To our knowledge, there is no other known specific, receptor-mediated inhibition of any of the other enzymes involved in porphyrin synthesis. Therefore, we must conclude that the downstream build-up of porphyrins at some point simply prevents further synthesis via the enzymes.

Another important finding was the time of plasma PPIX maxima. This was observed at 8 h, which was later than our in Vivo experiments, in which maximal brain tumor fluorescence was observed at 6 h^7^ with lower levels at 3 and 9 h. Similar time frames were observed in skin (maximum between 6.5 and 9.8 h)^22^ and blood cells (between 4 and 8 h).^35^ Although attempts at extrapolating plasma levels to tumor tissue in the brain are speculative, they are supported by observations in renal and bladder cancer. Inoue et al (2103)^36^ and Ishizuka et al (2011)^37^ find that only patients with tumor showed increases of plasma PPIX in response to 5-ALA administration. This indicates that plasma porphyrins originate in the tumor. Measuring porphyrins within malignant gliomas tissue would have been a preferable way for assessing the kinetics of tumor porphyrins, but any such measurements over hours in patients would not have been wrought with insurmountable hurdles.

Overall, however, we believe that our observation of peak PPIX levels at 8 h after administration suggests that 5-ALA might be given earlier than previously recommended, ie, 4 to 5 h prior to anesthesia.

Finally, peak ALA concentrations in plasma were observed within 1 h, falling rapidly thereafter. This observation corroborates that 5-ALA, a small molecule with 131 daltons, is rapidly absorbed from the digestive tract. This observation mitigates concerns regarding the application of 5-ALA in significant volumes of tap water 3 h prior to anesthesia and questions any necessity for an intravenous application. Others have found peak concentrations at 30 min using 40 mg/kg.^22^

### Histology and Fluorescence

The present study confirms the high positive predictive value of fluorescence^3,4,8,9,17,25,38,39^ revealing infiltrating tumor of a cell density as low as 25%. However, heterogeneity was marked, as described by others,^15,30,31^ suggesting factors apart from cell density play a role as well, eg, proliferation.^25,30,32,38^

### Differences between Spectrographical and Subjective Fluorescence Observation

We observed a strong relationship between visually perceived and spectrographical fluorescence, as previously described.^9^ However, there was also a degree of discordance between spectrographical fluorescence and the surgeon's visual impression with the surgical microscope as to whether he was confronted with no, weak, or strong fluorescence. This observation is related to the many factors that determine macroscopic fluorescence perception, which would be expected to differ from the more punctual, localized fluorescence values gained from spectrometry or extraction techniques.^30,32,25^

The observation cautions that basic studies on the mechanisms of fluorescence accumulation or microscopic fluorescence localization should utilize objective measurement techniques such as spectrometry or porphyrin extraction. On the other hand, when the emphasis of a study is on fluorescence-guided resections on a macroscopic level, such studies should acknowledge the confounders involved in identifying fluorescence and utilize subjective measures of fluorescence, as identified by the surgeon using the surgical microscope.

### Limitations

From the ex post perspective, we can question whether randomization for this study was really necessary. Randomization would not have been required for assessing pharmacokinetics, and our results regarding the subjective assessments of fluorescence of the surgeon were very clear-cut. However, when designing this study, we found it ethically more acceptable for patients and participating surgeons if patients were not knowingly assigned to lower doses than previously published as being efficacious. In addition, the surgeons’ assessments of the magnitude and distribution of fluorescence may appear somewhat crude and less objective than spectrography. Nevertheless, surgeons’ perception might differ from our spectrographical measurements, which were limited to few very localized measurements, in contrast to the more global view available to the surgeon.

## CONCLUSION

In conclusion, this study shows a positive dose–efficacy relationship with 20 mg/kg b.w. determined to be the most useful dose, both from a subjective and objective perspective. Pharmacokinetically, 5-ALA was rapidly absorbed and eliminated, with high bioavailability. On the other hand, a 10-fold increase in ALA resulted in only 4-fold higher contrast ratios between fluorescing tumor and adjacent tissue, questioning the efficacy of higher ALA doses for increasing useful fluorescence.

### Disclosures

This study was sponsored by Medac, Gesellschaft für klinische Spezialpräparate mbH. Wedel. U. Pichlmeier is a biometrician affiliated with this company. Walter Stummer has received lecture fees from Medac, Wedel, and Carl Zeiss Oberkochen.

## Supplementary Material

Supplemental material **Supplemental digital content** is available for this article at www.neurosurgery-online.com.

## Notes

**Supplemental digital content** is available for this article at www.neurosurgery-online.com.
