# Supplementary material for: Randomized, Prospective Double-Blinded Study Comparing 3 Different Doses of 5-Aminolevulinic Acid for Fluorescence-Guided Resections of Malignant Gliomas
Source: Neurosurgery. 2017 Apr 1;81(2):230–9. doi: 10.1093/neuros/nyx074 (PMC5808499; doi:10.1093/neuros/nyx074)

***Supplementary materials I: Statistical procedures***

Sample size: The confirmatory biometric analysis comprised the detection of a monotone dose-efficacy relationship between the amount of 5-ALA used and the fluorescence extent and quality within the tumor core. For a simplified sample size estimation, a binomial model with two attributive intensities/ qualities of the target variables (complete vs. incomplete fluorescence in the tumor core) was chosen. It was assumed that at the low dose level not more than 10% of the patients will show a complete fluorescence extent/quality in the tumor core whereas this will be achieved by 50% at the middle dose level and by 90% of the patients at the highest dose level. Using a trend test for comparing three-ordered binomial populations with a one-sided type 1 error of 5% and an individual power of at least 90%, a total of n=7 evaluable patients per dose level were planned.

Biometric analysis: All of the data recorded in the case report forms describing the sample, efficacy and safety were first analysed descriptively. The arithmetic mean and standard deviations as well as the robust measures of central tendency and dispersion such as the minimum, 1st quartile, median, 3rd quartile and maximum were reported for the continuous variables. The nominal and ordinal attributes were analysed by reporting the absolute and relative frequencies. Free text entries were compiled in text files. The homogeneity of the treatment arms with regard to demographic variables and baseline characteristics were reported descriptively. Single-tailed confirmatory tests on a (multiple) significance level of 5% were performed for the primary endpoints. Since both the treatment groups and the ordinal scaled endpoints have a natural order, the nonparametric Jonckheere-Terpstra test was used in the exact version to detect a monotone dose-efficacy relationship. This tests the null hypothesis (different dose levels produce the same fluorescence extents/qualities) versus the alternative hypothesis of a monotone non-falling dose-efficacy relationship (a higher dose of 5-ALA enhances fluorescence). Paired comparisons between the individual dose groups were not the focus of interest. Nevertheless, if the result of the global tests was significant, paired comparisons of the dose groups were performed at the local one-sided alpha-level 5%. This test strategy maintained the multiple alpha-level.

Apart from the global assessment of fluorescence intensity and quality, the fluorescence quality in the tumor core, tumor margin and normal tissue was assessed subjectively in specific areas and measured spectrometrically. Apart from analysis of the normalised peak heights, the ratio of the PPIX peak height of the histologically verified tumor tissue to that of the histologically verified normal tissue was used to describe the contrast optimum. To check the relationship between subjective fluorescence assessments and the parameters measured spectrometrically, correlation coefficients were calculated. Nonparametric tests for the increase in fluorescence intensity with the increase in subjective fluorescence quality would provide statistical proof of the objectification of the subjective assessments. In addition, a patient-specific analysis was performed. This was done by aggregating the spectrometric data per patient according to tissue type and plotting them as a function of dose. Measures of location and dispersion as well as nonparametric statistical methods (trend tests) served as exploratory tools to confirm the dose-dependent relationship. Statistical models such as regression and variance analysis could be used to describe the dose-efficacy relationship mathematically. Safety was assessed descriptively, stratified by dose on the basis of the standardised toxicity criteria. For analysis of laboratory parameters 95% confidence limits for the expected values were given. The study was analysed using an intent-to-treat approach for the primary endpoint.

Statistical analysis for Pharmacokinetics: The plasma concentration data for 5-ALA and PPIX and the pharmacokinetic parameters calculated from them were subjected to statistical analysis. Arithmetic means, standard deviations, geometric means, dispersion factors (geometric standard deviations), minimum and maximum values as well as the medians were listed. A frequency distribution was calculated for t_max_. Mean values were calculated only if at least two-thirds of the concentrations were above the lower limit of quantification (LOQ). LOQ/2 was used to calculate values below the LOQ. The AUC values for the different dose levels were checked for dose proportionality.

***Supplemental II: Toxicology***

*Adverse events (AE) and severe adverse events (SAE):*No deaths occurred during 28 days of follow-up. Two patients (29%) were reported with SAE in the 0.2 mg/kg b.w. group (subgaleatic hematoma, pneumonia), one patient (14%) in the 2 mg/kg group (excessive bleeding from skin incision) and one (14%) in the 20 mg/kg high dose treatment group (mild meningitis). None of the SAEs were reported to be drug related.

The incidence of AE was similar for all groups(6/7, 6/7, and 7/7 of patients), the most frequently being assigned to the CTC-category "Neurologic" ( speech impairment, headache, neuro-motor), followed by "Cardiac" (hypertension).

*Laboratory investigations:* Overall, there were no significant differences in any hematology parameter (including differential blood count), electrolytes, renal function parameters or coagulation parameters. A transient increase was found for the liver enzymes gamma-GT and the transaminases ALT/GPTand AST/GOT for between 14% and 57% of patients. The effect was seen most prominently in the highest dose group (20 mg/kg b.w.). **No significant elevation of gamma-GT was observed in the the two lower dose groups (2 and 0.2 mg/kg b.w.). However, marked increases in ALT/GPT and AST/GOT were also observed in the low dose groups during the first ten days after surgery, although not as high as for the 20mg/kg b.w. group.**

The clinical and neurologic condition of patients as determined by the Karnofsky Performance Scale (KPS) and NIH-stroke score did not change significantly after surgery. Changes in ECG parameters were not consistent and of unspecific nature. Median/individual supine systolic and diastolic blood pressure as well as pulse rate did not change in a clinical relevant manner comparing values between treatment groups (Fig. 8).

Fig. 8: Blood pressure course (mean ± SD) after administration of 5-ALA at three different doses. No significant differences in blood pressure were noted between treatment groups.


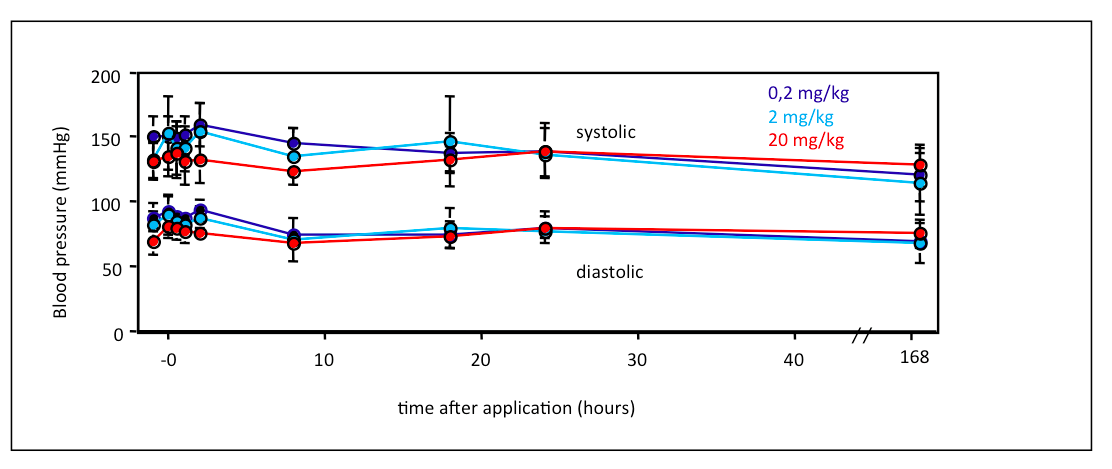

Supplement: Supplemental material — Supplemental digital content is available for this article at www.neurosurgery-online.com. [file nyx074_supp.docx]
